# A new methodology for sampling blackflies for the entomological surveillance of onchocerciasis in Brazil

**DOI:** 10.1371/journal.pone.0179754

**Published:** 2017-07-20

**Authors:** Érika S. do Nascimento-Carvalho, Raquel de Andrade Cesário, Vladimir Fazito do Vale, Arion Tulio Aranda, Ana Carolina dos Santos Valente, Marilza Maia-Herzog

**Affiliations:** 1 Laboratório de Simulídeos e Oncocercose, Oswaldo Cruz Institute, Oswaldo Cruz Foundation, Rio de Janeiro, Rio de Janeiro, Brazil; 2 Programa de Pós-Graduação em Biodiversidade e Saúde, Oswaldo Cruz Institute, Oswaldo Cruz Foundation, Rio de Janeiro, Rio de Janeiro, Brazil; 3 Onchocerciasis Elimination Program for the Americas, Guatemala City, Guatemala; George Washington University School of Medicine and Health Sciences, UNITED STATES

## Abstract

The effectiveness of the MosqTent^®^ trap was evaluated in endemic area to onchocerciasis in Brazil. This study seeks to provide subsidies for the monitoring of the onchocerciasis transmission in the country. The study was carried out at the Homoxi and Thirei villages, located in the Yanomami Indigenous Land, in the state of Roraima. This area presents hyperendemicity, high blackflies densities, large population migrations and mining activities. The Homoxi and Thirei villages are assisted by the Brazilian Ministry of Health. To conduct the present study, the village leader, health leaders and the Brazilian Ethics Committee were consulted. Blackflies captures were carried out simultaneously at the Homoxi and Thirei, using systematized methods to allow for comparisons between the traditional Human Landing Catch (HLC) and HLC protected by the MosqTent^®^. The female blackflies were captured at two equidistant capture stations per locality, by two collectors per station, for five consecutive days. Individuals captured by interval/station/day were counted, identified and maintained at -20°C. The underlying probability distributions and the differences between the methods for the independent sample data were verified in a comparative statistical analysis between the use of the MosqTent^®^ and the HLC. A total of 10,855 antropophilic blackflies were captured by both methodologies. A total of 7,367 (67.87%) blackflies belonging to seven species were captured by MosqTent^®^ —*Simulium incrustatum s*.*l* (99.06%); *S*. *guianense s*.*l* (0.74%), *S*. *oyapockense s*.*l* (0.01%), *S*. *exiguum* (0.10%), *S*. *metallicum* (0.05%), *S*. *ochraceum* (0.03%) and *S*. *minusculum s*.*l* (0.01%). Moreover, 3,488 (32.14%) blackflies belonging to four species were captured by HLC—*S*. *incrustatum s*.*l* (98.33%); *S*. *guianense s*.*l* (1.38%), *S*. *oyapockense s*.*l* (0.26%) and *S*. *metallicum* (0.03%). The MosqTent^®^ was more effective and efficient when compared to HLC. When comparing total blackflies captured/day, the MosqTent^®^ was more efficient than HLC (p = 0.031) with a means of 799.4 blackflies/day versus 217.6 blackflies/day by HLC. The results demonstrated improved performance and high reliability of the MosqTent^®^ compared to the traditional HLC method.

## Introduction

Blackflies constitute a public health problem in Brazil, since they transmit *Onchocerca volvulus* and *Mansonella ozzardi*. Anthropophilic species are extremely voracious and have a marked presence in riverside, wild and rural regions. The afflicted human populations are commonly attacked by clouds of blackflies, and their painful bites, as well as pathogen-transmitting ability, can cause severe immunological reactions, such as intense pruritus, irritation and fever. Onchocerciasis, commonly known as ‘river blindness’, has four *Simulium* species as vectors in Brazil—*S*. *guianense*, *S*. *incrustatum*, *S*. *oyapockense* and *S*. *exiguum*[1]. Amongst other anthropophilic species found in the endemic area, *S*. *guianense* is the one with the best competency to be the primary vector of onchocerciasis, being a very common species with high anthropophilic habits. *S*. *incrustatum*, *S*. *oyapockense* and *S*. *exiguum* are considered secondary vectors.

Onchocerciasis has been a challenge for world health authorities regarding epidemiological surveillance history [2]. In Brazil, the disease was confirmed in the Yanomami Indigenous Land, situated in northern Roraima [3] and northern Amazonas [4,5]. Because it is a focal anthroposis due to cumulative parasitic loads, where humans are the definitive and exclusive hosts, this disease is strongly connected to human migration. Thus, the most accepted hypothesis regarding its origin in the Americas is its introduction by the slave traffic from Africa, between the 16^th^ and 19^th^ centuries [6, 7].

Onchocerciasis is a chronic endemic parasitic disease that affects communities in both rural and wild areas, where human populations are exposed to high densities of vector bites. *O*. *volvulus* mainly inhabits the dermis, where microfilariae are responsible for important symptoms such as oncodermatitis—xeroderma, edema, papules, lichenification or pseudocystitis, depigmentation of the pre-tibial and inguinal regions, loss of elasticity, itching, atrophy and lymphatic stasis [8,9]. However, in older infections, ocular onchocerciasis can also be observed, where microfilariae invade the ocular structures by migration and contiguity, compromising visual capacity due to microfilariae death, in the anterior chamber of the eye, causing alterations such as eyelid edema, iritis, punctate keratitis, scleroceretitis, chorioretinitis and amaurosis. The prevalence and intensity of microfilaremia increases significantly with exposure to the vector and, consequently, patient age, although no association was found with sex and intraocular pressure of the affected indigenous people [10,11].

Even though onchocerciasis is classified as an emerging and neglected disease, it has been within the priorities of the Brazilian sanitary authorities since 1991, as a signatory country of the Onchocerciasis Elimination Program for the Americas (Programa para la Eliminación de la Oncocercosis en las Américas—OEPA) [12]. The strategy adopted by Brazil has included interruption of transmission by mass administration of ivermectin (Mectizan^®^, donated by Merck & Co Inc), twice a year and, more recently, four times a year. The ivermectin ministration must have a therapeutic coverage of ≥85% of the eligible population in all endemic communities of the region, including hypoendemic areas for 10–15 years. Priority for semi-annual treatment is indicated where epidemiological data are not on track to achieve elimination by the target date [12].

The Brazilian Ministry of Health has faced many challenges related to onchocerciasis, due to its ecoepidemiology, intrinsic to the nature of the disease and transversal to intervention actions and control program monitoring, and these are across national borders of disease. In 2015, Brazil and the Bolivarian Republic of Venezuela treated about 20 thousand people with ivermectin, a microfilariacide that acts directly on the microfilariae [13].

Despite the difficulties, a project to optimize the interruption of onchocerciasis transmission has been proposed, ensuring the introduction of new strategies to intensify the collective treatment in places where transmission continues. These include the introduction of complementary treatment with doxycycline in relapsing patients observed during parasitological evaluations. Clinical studies with the doxycycline antibiotic (daily administration for 4–6 weeks) have provided proof-of-concept that depleting *O*. *volvulus* of its symbiotic bacterium *Wolbachia pipientis* results in permanent sterilization of the parasite (200mg/d for 4 weeks or 100mg/d for 5 weeks) and can also exert a macrofilaricidal effect (6 weeks 200mg/d) [14,15,16]. Doxycycline does not kill the microfilariae but reduces their ability to develop into infective stages within the blackflies vectors [17]. Doxycycline is included in the 2013 WHO Model list of Essential Medicines [18].

To monitor transmission status it is necessary to verify the seasonal condition of the disease during the high-density period of the *Simulium* vector, from August to March in the Brazilian endemic area. Thus, it is indispensable to conduct systematized captures of adult females of the vector species in the sentinel areas, through protocolized techniques and methodologies, by competent, sensitized and trained professionals as a way of guaranteeing the subsistence of seasonal samples and the quality of the results, indispensable parameters for onchocerciasis transmission monitoring [12,19].

One of these parameters is the entomological evaluation by O-150 PCR technique, to determine the level of infective stage of *O*. *volvulus* larvae (L3 stage) in female blackflies, where a minimum of 6,000 blackflies collected from the transmission zone must be tested and be negative to represent elimination of transmission. This metric ensures decision-making regarding the evaluation of biting rates and transmission potential. Therefore, entomological surveillance is indicative of disease control status [12,20].

Entomological surveillance is a technical-based tool that subsidizes indicator management and operationalization, since it quantifies and stratifies levels of spatial and temporal relationships among components of the onchocerciasis transmission chain, allowing for the monitoring of their fluctuations and interventions [21]. Thus, it is fundamental that the adopted technique and methodology minimize possible biases that may interfere in the quality of *O*. *volvulus* diagnosis results in the captured vectors.

The simulation method currently used in entomological surveillance has been discussed by health and human rights authorities, and Brazil is not isolated with regard to this issue. To advance the introduction of a technology that supports the entomological surveillance of onchocerciasis, driven by the possibility of minimizing the health risks of professionals and protect those who work in capturing potential *Simulium* vectors in onchocerciasis endemic areas, the hypothesis of using an ideal trap with human bait to obtain vector samples is postulated. This must show the accuracy and profitability necessary for entomological surveillance processes, but without exposing the human, collector or bait, to the risk of being bitten by blackflies and other insects that can perform anthropophagy at the moment of capture, or the risk of acquiring diseases because of this activity. Therefore, the present study consisted in the evaluation of the effectiveness and efficiency of the MosqTent^®^ trap technology in the entomological surveillance of onchocerciasis vectors in Brazil.

## Materials and methods

### Study area

**Fig 1:**
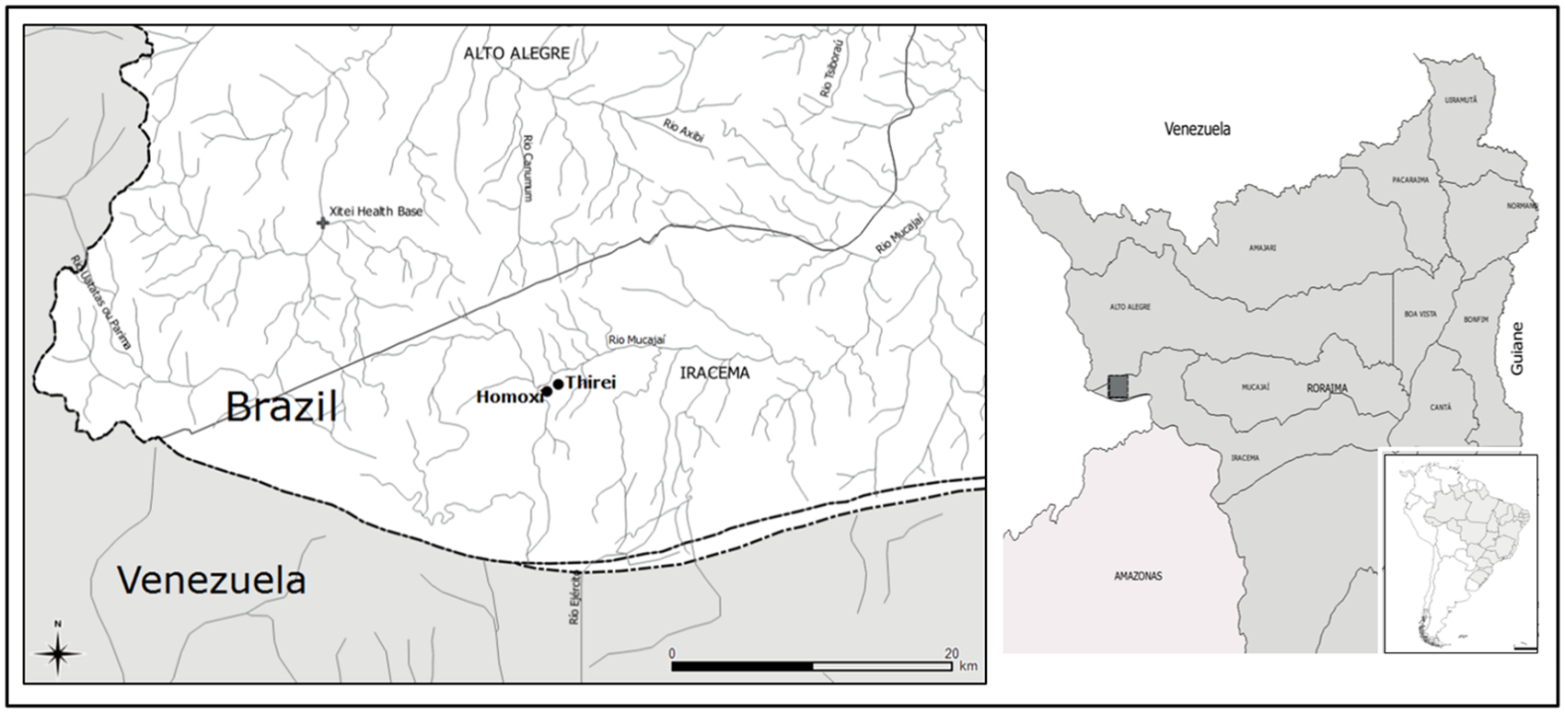
Map of the studied area, part of the sentinel area for onchocerciasis and located at the Brazil/Venezuela border.

The study was carried out in the Brazilian hyperendemic area of onchocerciasis, at Homoxi and Thirei villages, located in the Yanomami Indigenous Territory, in the Iracema municipality, located in the state of Roraima. Homoxi village is situated in the highlands (02°29'51"N 63°43'49"W|778m altitude) of the Serra do Parima, in the upper Mucajaí River, at approximately 30 km from Xitei Village in the municipality of Alto Alegre, which is a sentinel area for onchocerciasis and located at the Brazil/Venezuela border (Fig 1). This area features a high blackflies vector density and large migrations of the local population, in addition to being the most devastated by gold mining activities [22]. Homoxi Village originated from Xitei Village (dating back to the 1950s), formed by the migratory trajectory of the Thirei and Xere-ú population groups, which occupied a complex series of sites until reaching their current locations [23]. The Homoxi and Thirei Village population is assisted by the Brazilian Ministry of Health.

In order to conduct this study, the local leaders of health attention (called Tuchaua) were consulted. The catch areas were determined considering the dwellings and proximity to the Mucajaí River or its tributaries (probable *Simulium* breeding areas). Thus, **Homoxi** locality presents less dense vegetation followed by more conserved forest starting at a 200 m radius, flanking a flight lane. **Thirei** locality (02°30'07"N 63°43'24"W|768m altitude), on the other hand, is characterized by the presence of scrub or burned vegetation for family farming, with undergrowth and clearings in the less dense forest, followed by large areas cleared by mining activities. The Homoxi population passes through Thirei as a route to their daily activities.

### Experimental design

**Fig 2:**
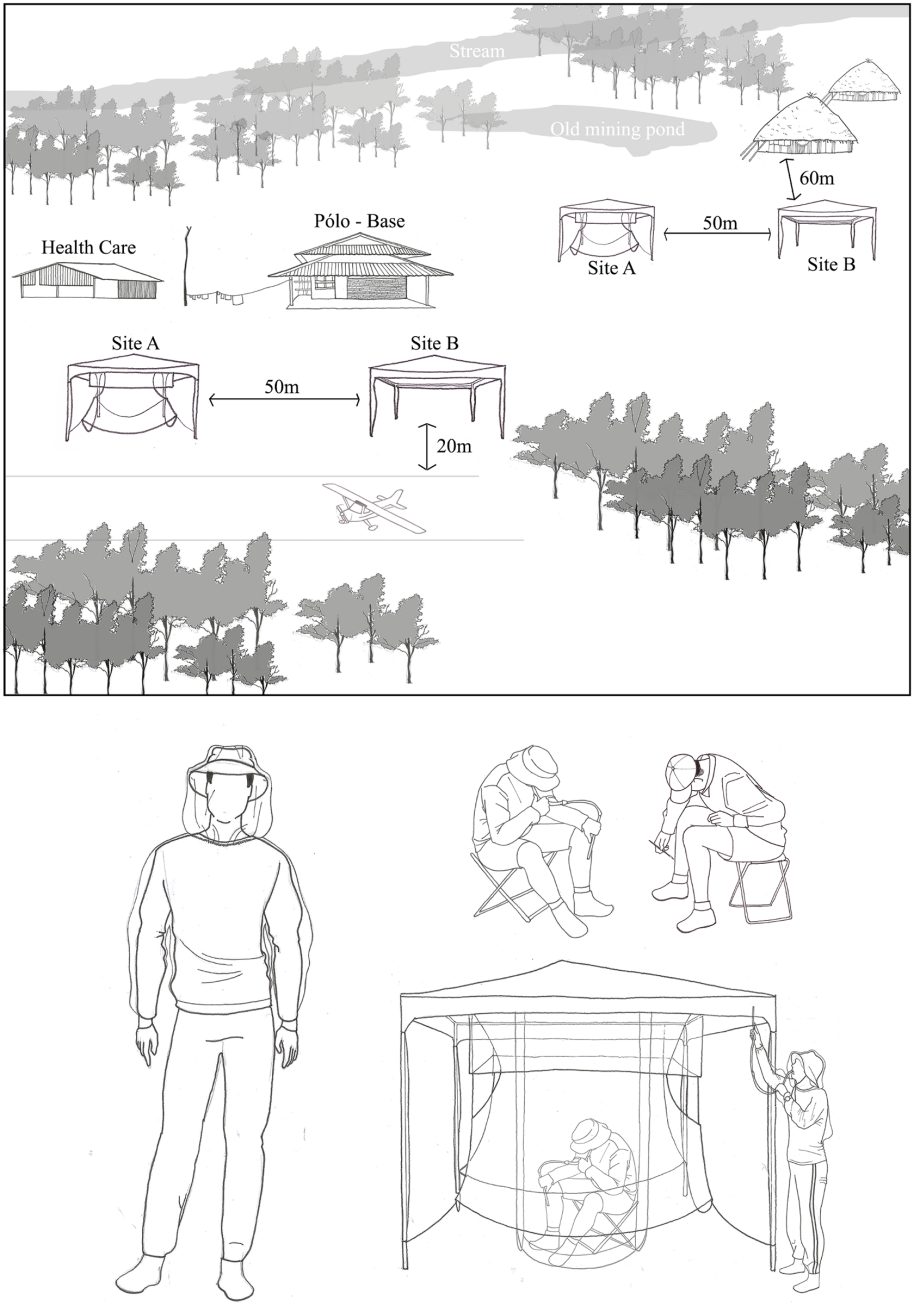
Experimental design—Scheme of the collection sites and surroundings.

Aiming to compare the Human Landing Catch (HLC) and the HLC protected by the **MosqTent**^**®**^ Trap [Process INPI-IPAS009-N ° 906941121, Deposit 26/10/2013] in the capture of anthropophilic blackflies, four capture stations were established considering the ecoepidemiological characteristics for onchocerciasis vectors. Two capture stations were located at **Homoxi** and two at **Thirei**, arranged at a 50 m equidistance at each of the selected areas (Fig 2). To maximize the chance that the blackflies inside the trap were seeking a blood meal instead of just being curious, capture stations were located at least 100m away from the breeding site.

The MosqTent^®^ Trap has an innovative design and measures 2.40 x 2.00 x 1.90m [24]. It is composed of a white multi-milled polyester mesh, with a human bait lock chamber protected in the center of the trap, secured by a mooring to a white tent (gazebo) 3 x 3 x 2.5m in height (Fig 2).

The capture were carried out in parallel by two collectors at each capture station, during five consecutive days in May 2016 (25^th^ to 29^th^), in the morning (07:00–11:50h) and in the afternoon (13:00–17:50h), with 50-minute catch intervals and 10-minute pauses, totaling 10 hours of capture/day/two collectors/station. The methods were systematized to allow for comparative analyses between the HLC and MosqTent^®^ methodologies, since both were evaluated simultaneously. To minimize interference between the methods, the positions of the capture technicians were changed daily, avoiding positional bias and decreasing the bait effect per individual. This arrangement was designed to allow *Simulium* vectors to choose between HLC and the MosqTent^®^, placing the methods in an equal supply position in the process.

### Sample processing

The blackflies captured were conserved in absolute ethyl alcohol PA, separated by interval and capture stations. All blackflies were counted, identified and conditioned at -20°C, after separation from the other insects captured during the experiment. Specific identification was based on morphological characters using a stereoscopic microscope and a dichotomous key [1]. All sample data were recorded on LSO/IOC-Fiocruz (Laboratório de Simulídeos e Oncocercose/Oswaldo Cruz Institute, Oswaldo Cruz Foundation) field and triage/identification records.

### Statistical analyses

The GraphPad Prism version 7 software package (GraphPad Software, La Jolla, CA, www.graphpad.com) was used for data analyses. The blackflies captured at each station/day were quantified by species and their abundances were measured. The Kolmogorov-Smirnov Test was used to determine if the underlying probability distributions of the HLC and MosqTent^®^ methods differed significantly from each other. The Mann-Whitney test was used to evaluate statistical differences between both methods, as appropriate for data from independent samples from the same population.

### Ethics statement

All research protocols and procedures involving HLC and the MosqTent^®^ Trap were reviewed and approved by the Ethics Committee of Brazil, CEP/FIOCRUZ/IOC-CAAE-N°54375016.5.0000.5248. All experiments were performed by trained volunteers who read and signed required informed consent documentation. We also emphasize that the team that participated in the fieldwork was treated with ivermectin one week before performing the captures.

## Results

A total of 10,855 female blackflies were captured by both methodologies after five days. With the MosqTent^®^, 7,367 (67.87%) blackflies were captured, belonging to seven anthropophilic species—*Simulium incrustatum s*.*l* being the most abundant (99.06%); *S*. *guianense s*.*l* (0.74%), *S*. *oyapockense s*.*l* (0.01%), *S*. *exiguum* (0.10%), *S*. *metallicum* (0.05%), *S*. *ochraceum* (0.03%) and *S*. *minusculum s*.*l* (0.01%). Moreover, with the HLC method, 3,488 (32.14%) blackflies were captured, belonging to four anthropophilic species—*S*. *incrustatum s*.*l* (98.33%); *S*. *guianense s*.*l* (1.38%), *S*. *oyapockense s*.*l* (0.26%) and *S*. *metallicum* (0.03%) (Table 1).

**Table 1:** *Simulium* species captured during the five days of the experiment with MosqTent^®^ trap and HLC method, at Homoxi and Thirei sites, Roraima/Brazil, in May 2016.

SPECIES	MOSQTENT^®^						HLC						TOTAL (%)
	HO	(%)	TH	(%)	HO+TH	(%)	HO	(%)	TH	(%)	HO+TH	(%)	
*Simulium incrustatum sl*	3970	(99.34)	3328	(98.80)	7298	(99.06)	1060	(97.43)	2370	(98.75)	3430	(98.33)	10728 (98.83)
*Simulium guianense sl*	19	(0.47)	34	(1.00)	53	(0.74)	24	(2.21)	24	(1.00)	48	(1.38)	101 (0.93)
*Simulium oyapockense sl*	1	(0.02)	0	(0.00)	1	(0.01)	3	(0.27)	6	(0.25)	9	(0.26)	10 (0.09)
Simulium exiguum*	5	(0.12)	3	(0.07)	8	(0.10)	0	0	0	0	0	0	8 (0.07)
*Simulium metallicum*	2	(0.05)	2	(0.05)	4	(0.05)	1	(0.09)	0	0	1	(0.03)	5 (0.04)
Simulium ochraceum*	0	(0.00)	2	(0.05)	2	(0.03)	0	0	0	0	0	0	2 (0.01)
Simulium minusculum sl*	0	(0.00)	1	(0.03)	1	(0.01)	0	0	0	0	0	0	1 (0.009)
**Total**	3997 (100)		3370 (100)		7367 (100)		1088 (100)		2400 (100)		3488 (100)		10855 (100)

HO: Homoxi; TH: Thirei; HLC: Human Landing Catch.

*Species that were not captured with HLC method.

**Fig 3:**
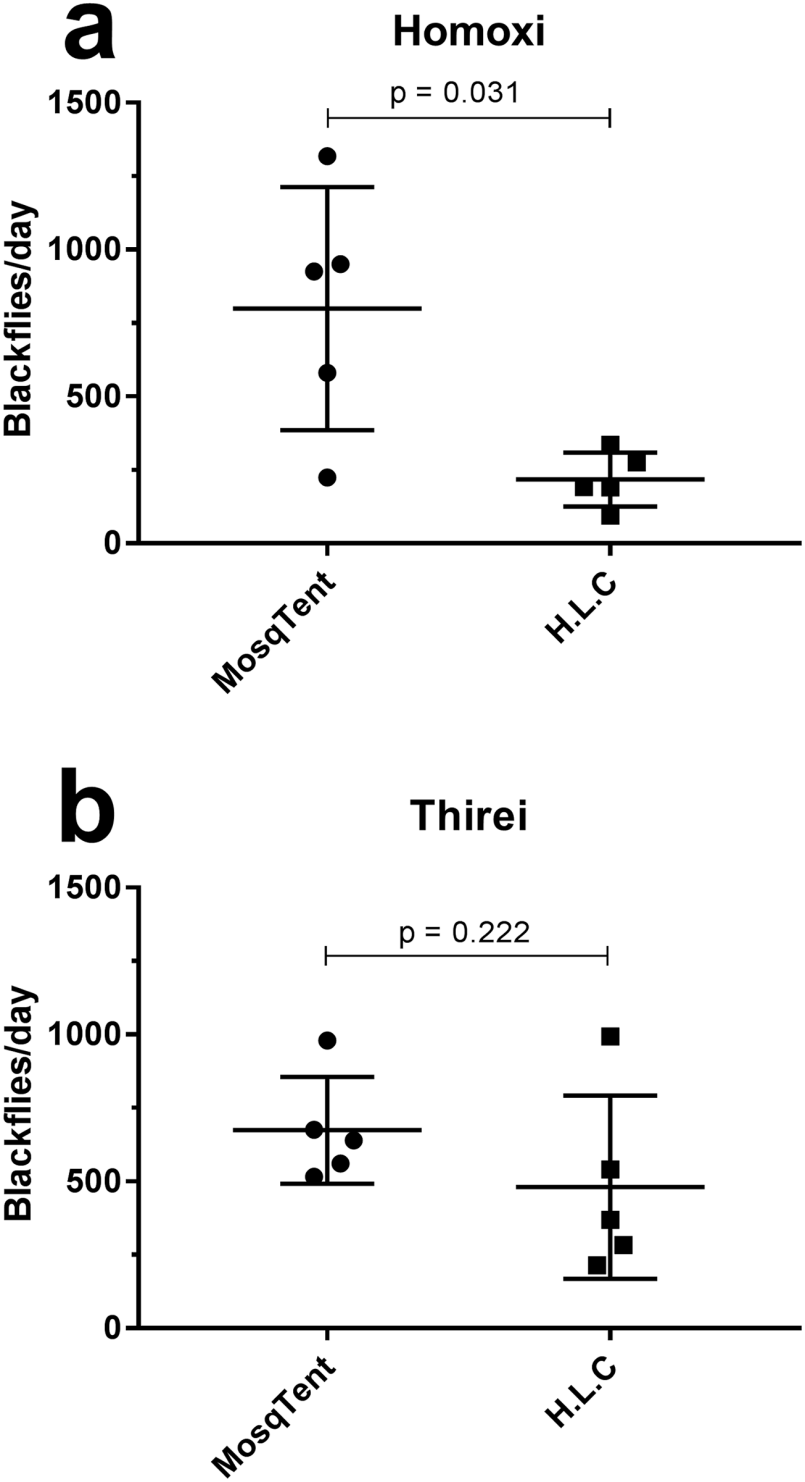
Simuliidae/day by the traditional HLC methodology at Homoxi (a) and Thirei (b).

When comparing the total amount of blackflies captured/day, the MosqTent^®^ was more efficient than the HLC method with a means of 799.4 blackflies/day against 217.6 blackflies/day by the traditional HLC methodology at Homoxi (Mann-Whitney, *p = 0.0317, U = 2, standard error of mean [MosqTent^®^] = 185.3, standard error of mean [HLC] = 41.2) (Fig 3a). On the other hand, no significant difference was observed between the amounts of captured insects per day when comparing both methodologies at Thirei (Mann-Whitney, ns p = 0.2222, U = 6, standard error of mean [MosqTent^®^] = 81.3; standard error of mean [HLC] = 139.4) (Fig 3b).

**Fig 4:**
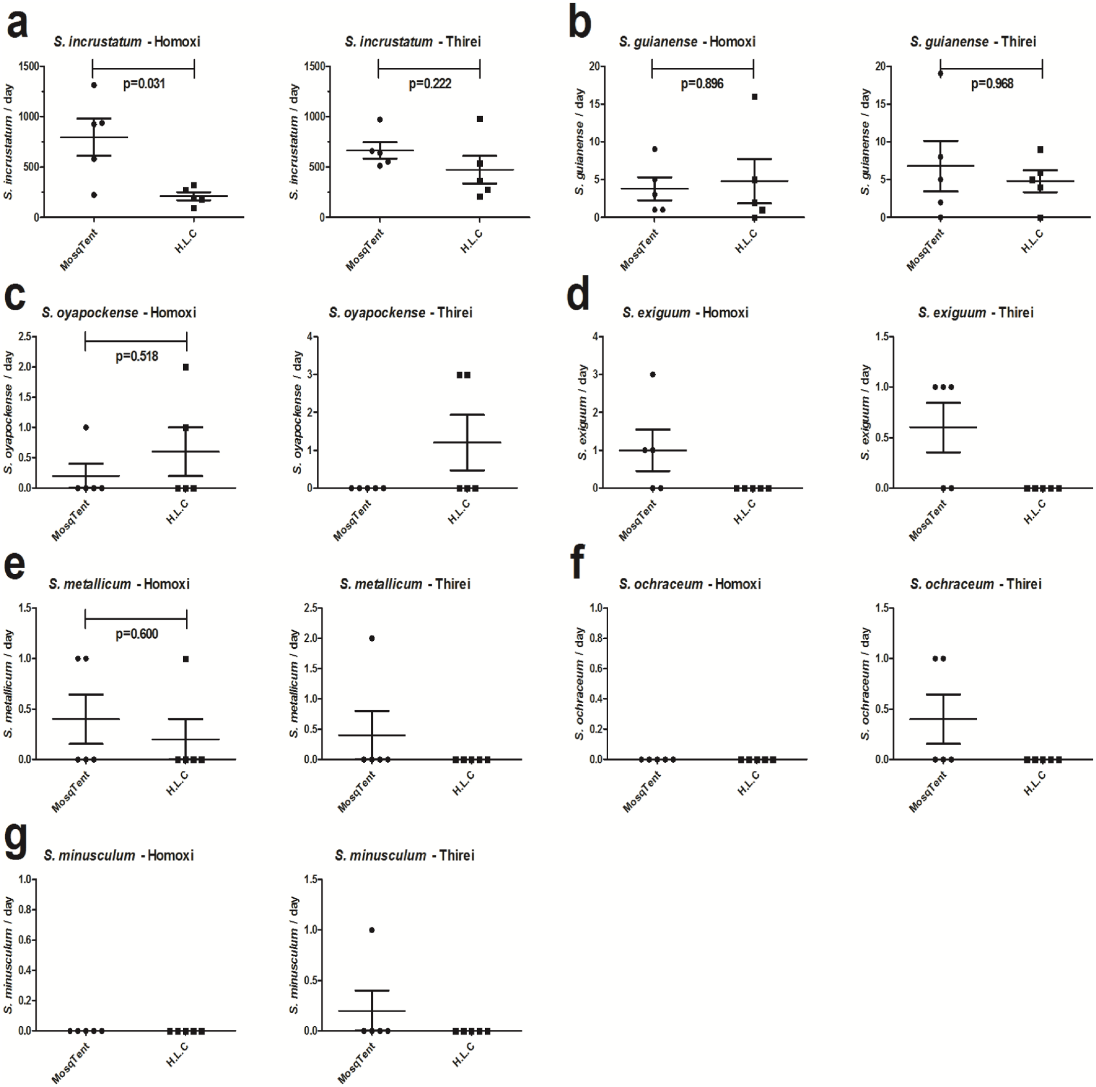
MosqTent^®^ trap performance and HLC method at Homoxi and Thirei.

**Fig 5:**
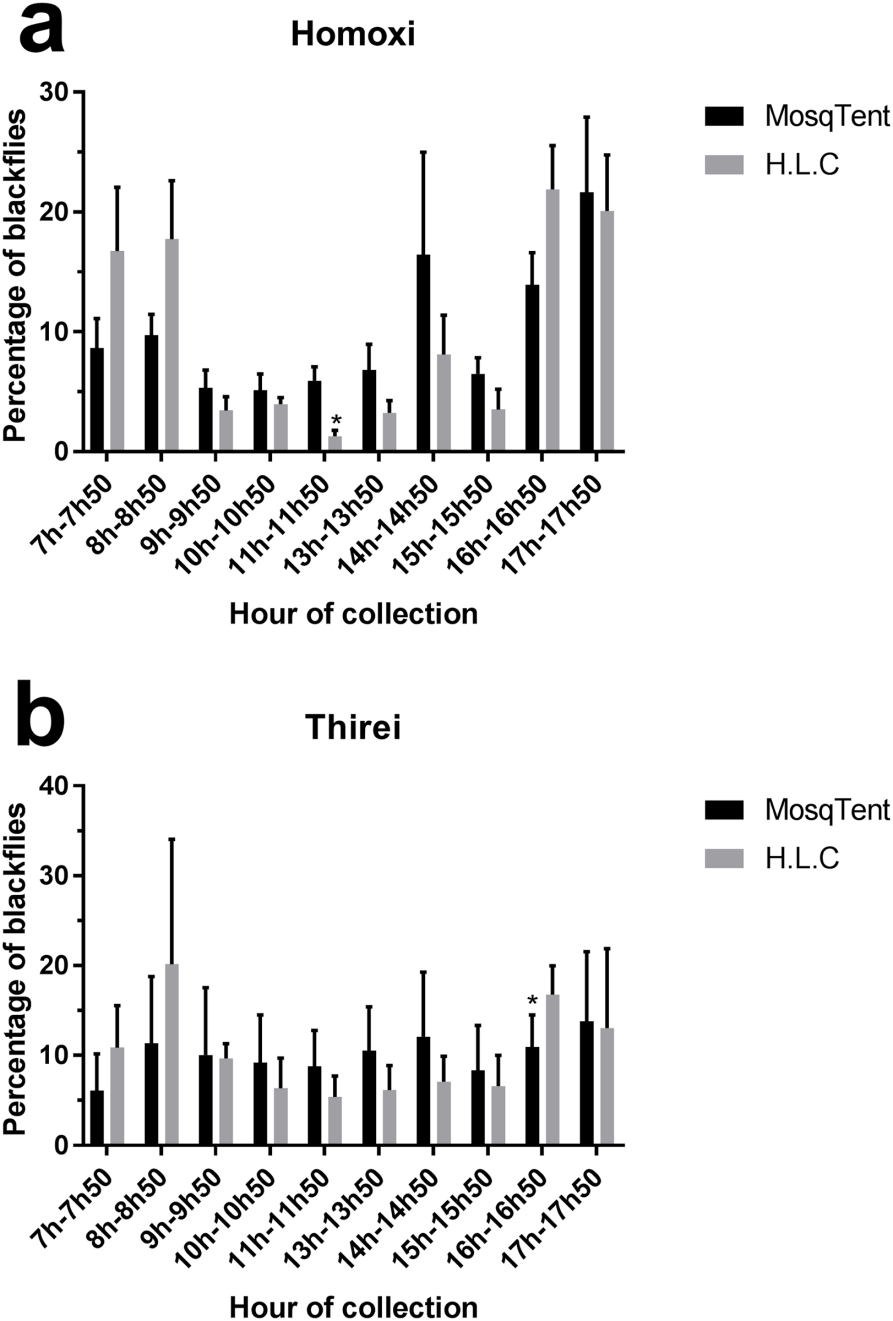
Bite activity of Simuliidae at Homoxi (a) and Thirei (b).

The MosqTent^®^ trap performance was similar to the HLC method at both Homoxi (Mann-Whitney, ns p = 0.9155, U = 11, standard error [MosqTent^®^] = 1.497, standard error [HLC] = 2.922) and Thirei (Mann-Whitney, ns p = 1.0000, U = 12, standard error [MosqTent^®^] = 3.338, standard [HLC] = 1.463) (Fig 4a–4g) regarding the number of captured individuals of the main vector, *S*. *guianense*, per day. Bite patterns during the day were analyzed separately at the two studied locations, considering all blackflies captured. At Homoxi, the bite activity of Simuliidae females showed a bimodal pattern, with one activity peak in the early morning (between 07:00 and 08:50 h) and one in the afternoon (between 16:00 and 17:50 h) (Fig 5a). At Thirei, the bite activity was homogeneously distributed throughout the day (Fig 5b). When comparing the anthropophilic profile of the blackflies between the MosqTent^®^ and HLC catches, results indicate that the percentage of captured species maintained similar seasonal fluctuation at both Homoxi and Thirei, except between 11:00–11:50 at Homoxi and between 16:00–16:50h at Thirei, where there was a difference between the capture methods.

## Discussion

One of the most productive ways to capture anthropophilic blackflies during the monitoring and epidemiological surveillance of onchocerciasis, as well as other vectors of human diseases, is the use of a human serving as bait [25,26]. However, the current "gold standard" for anthropophilic vector capture, HLC, although effective and an essential parameter for the calculation of entomological indices, such as biting rate, anthropophilic degree and human exposure, puts participants at risk for vector-borne diseases [27].

Above all, for ethical reasons and from a perspective of possible health risks to the capturing technician, the use of HLC is prohibited in Brazil, according to Brazilian Legislation—Resolution N° 466 of December 12, 2012 of the National Health Council. The use of technologies that facilitate the capture of anthropophilic blackflies, with both safety and quality, is essential in order to obtain critical data for the entomological surveillance of these vectors. The results found herein demonstrate the feasibility of using the MosqTent^®^ trap in capturing anthropophilic blackflies in an endemic onchocerciasis area in the Brazilian Amazon. This was proven a safe, efficient, sensitive and rigorous method to capture the human onchocerciasis vectors, as well as profitable and as widely deployable as HLC.

Since the transmission status of *O*. *volvulus* in an area is estimated mainly through the infectivity rate of the vectors, along with other entomological indices, it is essential to capture a large number of females, due to the requirements of the pool-screening technique [28]. Therefore, the search for efficient methodologies to capture vectors that eliminate or diminish the need for contact with the vectors themselves has been intense.

Traps with biological/chemical human bait are proven to be the most effective, probably because the bait emits many localization signals, with smell being one of the most powerful, since it is a combination of many molecules [29]. Thus, comparing the MosqTent^®^ and HLC trap technologies can contribute to the improvement of entomological surveillance of onchocerciasis in the country, especially in areas of low blackfly density and in the field of worker health, since this disease continues to be a major challenge for public health.

Encouraging results were obtained during this first performance evaluation of the MosqTent^®^ trap in the capture of anthropophilic blackflies, since the absolute richness of species collected by the MosqTent^®^ method was higher than when using HLC. In addition, while the performance of MosqTent^®^ was similar to the HLC method at Thirei, the MosqTent^®^ trap proved to be more efficient at Homoxi, capturing a 3-fold higher daily average of insects than the HLC method. These results suggest that factors other than the chemical attractants released by humans may contribute to blackflies capture when the MosqTent^®^ trap is used. It is possible that the contrast between the whitish coloration of the trap and the background landscape attracts these insects, since visual stimuli are recognized as important when blackflies are searching for blood [30,31].

In addition, it is important to note that all species captured with MosqTent^®^ have anthropophilic habit. The literature describes that to orient towards hosts, female detect a variety of chemical and physical cues including body odor, CO_2_, moisture, heat, visual contrast, temperature and phagoestimulants. Of these sensory cues, the ~4% CO_2_ exhaled in breath is a potent behavioral activator and attractant for females, and is often considered to be the most important sensory cue used by these disease vectors to find humans [32,33]. Another possible explanation would be that blackflies become trapped between the MosqTent^®^ trap wall screens, which facilitates their capture. The HLC method, however, depends exclusively on the agility of the capture technician, where loss of specimens is common between their landing in the search for blood and their capture by the technician.

The effectiveness of the MosqTent^®^ trap in the capture of *S*. *guianense*, was similar to the traditional method (HLC) at both sampling locations, despite the low *S*. *guianense* densities found during the study period (May). Results from previous studies show that high densities of the secondary vector, *S*. *incrustatum*, occur between March and July, for this reason May is within the suitable period for its capture and is not suitable for capturing *S*. *guianense*, because high *S*. *guianense* densities in this study area occur between August and December, corroborated in this study [34,35]. Considering the results found for all species captured in the area, mainly *S*. *incrustatum* (biting rate Homoxi [MosqTent^®^] = 743.12/day or 23036.61/period, [HLC] = 166.77/day or 5169.77/period; Thirei [MosqTent^®^] = 805.45/day or 24969.09/period, [HLC] = 488.02/day or 15128.59/period), indicate that MosqTent^®^ efficiency was not compromised. We emphasize that the results of this study demonstrate that MosqTent^®^ capture methodology will not interfere in the capture of 6,000 specimens of *S*. *guianense* required for the Onchocerciasis monitoring in the endemic area, according to WHO guidelines.

Although some developed blackflies capture methodologies have been tested in an attempt to substitute HLC [36,37,38] the only approach with the technical capacity to replace HLC is the MosqTent^®^. In comparison to recent reports in the literature, traps using adhesive-coated substrate to retain blackflies using organic CO_2_ and/or commercial synthetic attractants as bait do not allow for HLC replacement in cases of applications in entomological surveillance, since they do not allow for calculations of biting rates and comparative analyses with historical data. The advantage of the MosqTent^®^ for blackflies capture is that it does not require the use of organic CO_2_, and is allowing a more secure method for use in the wild and endemic onchocerciasis area in Brazil, since two people functions as bait, albeit protected. The method also does not mask the effectiveness or calculation of biting rate, and also maintains the physical integrity of the trap until the end of the capture event.

Another important index for the entomological surveillance of onchocerciasis is the parity rate of blackflies females. Therefore, it is necessary that the parity rate of the captured blackflies by alternative methods be similar to the parity rate obtained by the traditional HLC method. Although the present study did not evaluate parity rate, the data indicate that there is no difference in this parameter, since no significant difference was observed by Mann-Whitney test between the activity pattern of the females captured by the MosqTent^®^ and by HLC throughout the day. According to studies developed by Grillet [39] in Venezuela, parity *S*. *guianense* females exhibited a pattern of predominantly bimodal activity, while *S*. *incrustatum* and S. *oyapockense* exhibited predominantly unimodal activity, suggesting that the physiological state and reproductive cycles of these species determine the periodicity of the search for blood and our study supports the bimodal activity.

The quantification of the density of anthropophilic blackflies females in a given locality is traditionally performed by HLC, and through its application, bite rates (daily, monthly, seasonal and annual) are calculated [40], in addition to species, climatic condition, parity status (nulliparous and parity), location and host availability [41,42,43]. Considering that gonotrophic cycles are short and blood supply is followed by oviposition, this demonstrates that cohort frequency leads to an increase in the transmission potential of blackflies vectors [44]. Thus, the substitution of HLC by any other trap using synthetic bait implies in the formulation of a correction factor between the two methods, in view of the conducted comparison. As the MosqTent^®^ uses exclusively protected human bait, without any synthetic attractiveness, and mainly because no statistically significant differences were observed when compared to HLC, despite the number of females caught was higher with MosqTent^®^. It is presumed that there is no need to develop a correction factor for the bite rate calculations, since the only change in the method is the protection of the capture technician.

The MosqTent^®^ trap was proven efficient and effective in the capture of anthropophilic blackflies in the endemic onchocerciasis area in Brazil. In addition, 67.87% of the blackflies were captured, a higher number than that using the HLC method, of 32.14%. Maybe due to having staff collecting flies the entire time in the tent, with the similar collection time as the HLC, an attractive and a collector, as opposed to only 10 minutes per hour as tested in other cited publications. In this regard, some reports have been published on alternative capture methods, such as the Esperanza window trap, which proved effective but inefficient for the capture of *Simulium ochraceum*, an onchocerciasis vector in Mexico and Guatemala [38]. This method was also evaluated in the capture of blackflies vectors in Africa, presenting the same inefficient result for entomological surveillance [45]. Similarly, a human-baited tent tested to capture *Simulium damnosum* complex in Ghana also proved inefficient for surveillance [46].

Considering the urgency of the search for a capture method of possible vectors that does not use unprotected people as bait, the use of the MosqTent^®^ trap in the context of the Brazilian Onchocerciasis Elimination Program is pointed out herein as a viable alternative to the traditional HLC method. A study to evaluate the MosqTent^®^ trap in the different Brazilian phytogeographic regions is suggested, in order to verify its performance on different blackflies community structures.

## Conclusions

This pioneer study regarding blackflies vectors in a Brazilian endemic area will benefit a large number of people simultaneously, since the results observed herein will allow for national technological advances for workers, technical field and laboratory staff, concerning anthropophilic blackflies captures and vector diagnosis in the entomological surveillance of onchocerciasis. With the implementation of the MosqTent^®^, higher security levels will be present in Brazilian Ministry of Health entomological surveillance activities, since no exposure of the capture staff in the field is necessary. The results with the MosqTent^®^ trap demonstrated better performance and high reliability when compared to HLC, allowing for the capture a greater number of blackflies with the same effectiveness as this traditional method.
